# Sleeping

**Published:** 1882-06

**Authors:** 


### SLEEPING: OR, THIS AND THAT.

SOME one-sided, one-eyed individual has made the following calculation, which is mathematically correct, but practically as false and mischievous as can well be expressed in plain English.

### THIS.

"The difference between rising every morning at six and at eight, in the course of forty years, amounts to twenty-nine thousand three hundred and ninety hours, or three years, one hundred and twenty-one days and sixteen hours, which are equal to eight hours a day for exactly ten years; so that rising at six will be the same as if ten years of life were added, wherein we may command eight hours every day for the cultivation of our minds and the dispatch of business."

### THAT.

The following communication was recently made to a British society:

"A Chinese merchant had been convicted of murdering his wife, and was sentenced to die by being deprived of sleep. This painful mode of death was carried into execution under the following circumstances:

"The condemned was placed in prison under the care of three or the police-guard, who relieved each other every alternate hour, and who prevented the prisoner from falling asleep, night or day. He thus lived for nineteen days without enjoying any sleep. At the commencement of the eighth day his sufferings were so intense that he implored the authorities to grant him the blessed opportunity of being strangulated, guillotined, burned to death, drowned, garroted, shot, quartered, blown up with gunpowder, or put to death in any other conceivable way which their humanity or ferocity could invent."

Crazy people can't sleep at all. The man who is "fat as a fool," sleeps all the time, except when eating. A thin Yankee, so keen after making money that the look of his eye goes right through you, and his word pierces like a poignard, goes to bed at midnight and is ready for work or a bargain at peep of day, is teetotally dried up at the age of forty years; the skin fairly clings to his bones, and before you know it, he has evaporated. But look at that fat, lazy Dutchman, who never was fairly and fully awake since he was born, and, in spite of pipes of "lager," and whole tierces of tobacco, he lives to see the third, if not indeed the fourth, generation. Whoever heard of any one sleeping himself to death? But no sleep brings death in nineteen days.

It is stated of one of the most eminent, one of the very best of the men of this nineteenth century, that, while at college, he retired at twelve o'clock at night and rose at six and a half. A man of his activity of brain ought to have had at least eight hours' sound sleep in every twenty-four. But look at the sketch he makes of himself about this very time:

"Pardon the vagaries of a half-crazed student. My old complaint, the blues, has come upon me like a strong man armed. Misanthropy, I despise it, I loathe it, yet I hug it to my heart. Truly I am depressed, devoured by spleen, fostering a crabbed, morose, silly, churlish, sinful despondency."

Such feelings as these continued to haunt this gentleman more or less throughout life, and he failed to reach "three-score" by several years. That insufficient sleep would aggravate the malady of misanthropy, even when it does not largely aid in originating it, and that it lays the foundation for a premature death, no intelligent physician will deny. The observant reader need only bring to his remembrance the disagreeable feelings of any day which has been preceded by a sleepless night, to be fully convinced of the serious evils which must follow a systematic short allowance of sleep.

We heard one of the greatest theological minds of the century advise that time should not be wasted; that students should carry some book in the pocket which they could take out and read, when waiting at an appointment or for sitting down to a dinner, while others were collecting; that even if but half-a-dozen lines were read, it was that much time saved. He believed in his theory, put it in practice, died early and—demented! The brain must have rest; sleep is the best rest.

Let parents who do not want their children to die of water on the brain, allow them to have the fullest amount of undisturbed sleep they possibly can take, especially while at school.

The more sick people can sleep, the sooner they will get well. Sleeping in the daytime, if before noon, enables them to sleep better the following night. Students, women, and nervous persons need all the sleep they can get, and so do the melancholy and those who are in trouble. Early rising is not condemned, it is heartily commended. But if not preceded by an early retiring, it is a crime against the body.

